# Obstructive sleep apnea and the risk of gout: a population-based case-control study

**DOI:** 10.1186/s13075-020-02176-1

**Published:** 2020-04-25

**Authors:** Caroline van Durme, Bart Spaetgens, Johanna Driessen, Johannes Nielen, Manuel Sastry, Annelies Boonen, Frank de Vries

**Affiliations:** 1 Department of Internal Medicine, Maastricht University Medical Centre+, Maastricht, The Netherlands; 2 Department of the Musculoskeletal System, Centre Hospitalier Chrétien, Liège, Belgium; 3 Department of Clinical Pharmacy and Toxicology, Maastricht University Medical Centre+, Maastricht, The Netherlands; 4 CAPHRI, Care and Public Health Research Institute, Maastricht University, Maastricht, The Netherlands; 5 Division of Pharmacoepidemiology and Clinical Pharmacology, Utrecht Institute for Pharmaceutical Sciences, Utrecht University, PO Box 80082, 3508 TB Utrecht, The Netherlands; 6 NUTRIM School of Nutrition and Translational Research in Metabolism, Maastricht University Medical Center+, Maastricht, The Netherlands; 7 Academic Sleep Center CIRO, Horn, The Netherlands; 8 MRC Lifecourse Epidemiology Unit, University of Southampton, Southampton General Hospital, Southampton, UK

**Keywords:** Gout, Obstructive sleep apnea (OSA), Case-control study, Comorbidity

## Abstract

**Background:**

Patients with obstructive sleep apnea (OSA) might be at risk of gout because of pathophysiological mechanisms that can lead to hyperuricemia and eventually gout or because of shared risk factors between both diseases. The objective of the present study was to investigate the risk of gout in patients with OSA.

**Methods:**

A population-based case-control study using the UK Clinical Practice Research Datalink GOLD including all patients aged 40 years and older with a first diagnosis of gout between 1987 and 2014. Gout cases were matched by year of birth, sex, and practice to non-gout controls. Conditional logistic regression estimated the risk of gout with an earlier diagnosis of OSA. Analyses were adjusted for lifestyle factors, comorbidities, and recent drug use.

**Results:**

One hundred eleven thousand five hundred nine cases were matched with 210,241 controls. Patients with OSA were at increased risk of gout (OR 1.86; 95%CI (1.71–2.02). However, this association disappeared (OR 1.05; 95% CI 0.96–1.16) after adjustment for smoking status, body mass index (BMI), alcohol use, a history of heart failure, diabetes mellitus, renal function, and recent use of diuretics and other medications. Among females with OSA and patients with OSA associated with heart failure, renal impairment, or higher BMI, the risk of gout was however still increased when compared to the total control population.

**Conclusion:**

This study showed that the observed association between OSA and gout disappeared after adjustment.

## Introduction

Gout is the most common inflammatory arthritis [1], affecting up to 1–2% of adults, and leads to disability and reduced quality of life [2]. Gout is characterized by the deposition of monosodium urate (MSU) crystals in synovial fluids and other tissues. Individuals suffering from gout often have a complex profile of comorbidities, including cardiovascular disease, diabetes mellitus, and kidney disease [3]. One of the comorbidities in gout that has received more attention over the past years is obstructive sleep apnea (OSA) [4]. Various underlying mechanisms may explain an association between gout and OSA. First, OSA-induced hypoxemia causes a rise in adenosine triphosphate (ATP) degradation which eventually increases purine concentrations and their end product uric acid [5]. Second, hypercapnia and acidosis caused by OSA could influence the likelihood of MSU precipitation [6]. Third, excretion of lactic acid, generated during the hypoxic episodes in OSA, could result in a higher renal reabsorption of uric acid [7].

Alternatively, the relationship could also be explained by shared risk factors of gout and OSA, such as age, obesity, metabolic syndrome, renal impairment, and heart failure [8].

Two prospective studies in large United Kingdom (UK) primary care databases have demonstrated a 1.5-fold increased risk of developing gout among patients with OSA [4, 9] with the overall risk peaking 1 to 2 years after OSA diagnosis [9]. While both papers statistically adjusted their analyses for body mass index (BMI), type 2 diabetes mellitus, ischemic heart disease, hypertension, the use of diuretics of an unspecified class, and alcohol consumption, renal impairment was either ignored or considerably under-recorded [4]. Under-recording could be explained by selecting only medical diagnoses of chronic kidney disease (CKD), not taking > 30 million records of estimated glomerular filtration (eGFR) rates into consideration (that are available as of 2018) [4]. As CKD is a well-known risk factor for gout [10, 11], adequate statistical adjustment for this risk factor is important. Furthermore, both studies ignored the presence of heart failure, which is associated with both gout [12] and with sleep disorders, especially OSA [13].

The objective of the present study was to investigate the risk of gout in patients with OSA, while accounting for all relevant potential confounders, including CKD and heart failure.

## Methods

### Data source

Data for the present study were obtained from the Clinical Practice Research Datalink (CPRD) in the UK, previously known as the General Practice Research Database (http://www.cprd.com). CPRD contains the computerized medical records of approximately 13 million patients under the care of general practitioners (GPs) in the UK, representing 6.9% of the total UK population [14]. Practices contribute to CPRD only if their data quality meets research standards. Since 1987, data recorded in the CPRD include demographic information, prescription details, lifestyle parameters, clinical events, preventive care provided, and specialist referrals. CPRD has been extensively validated [15] and has been previously used to study gout [16].

### Study population

**Fig. 1:**
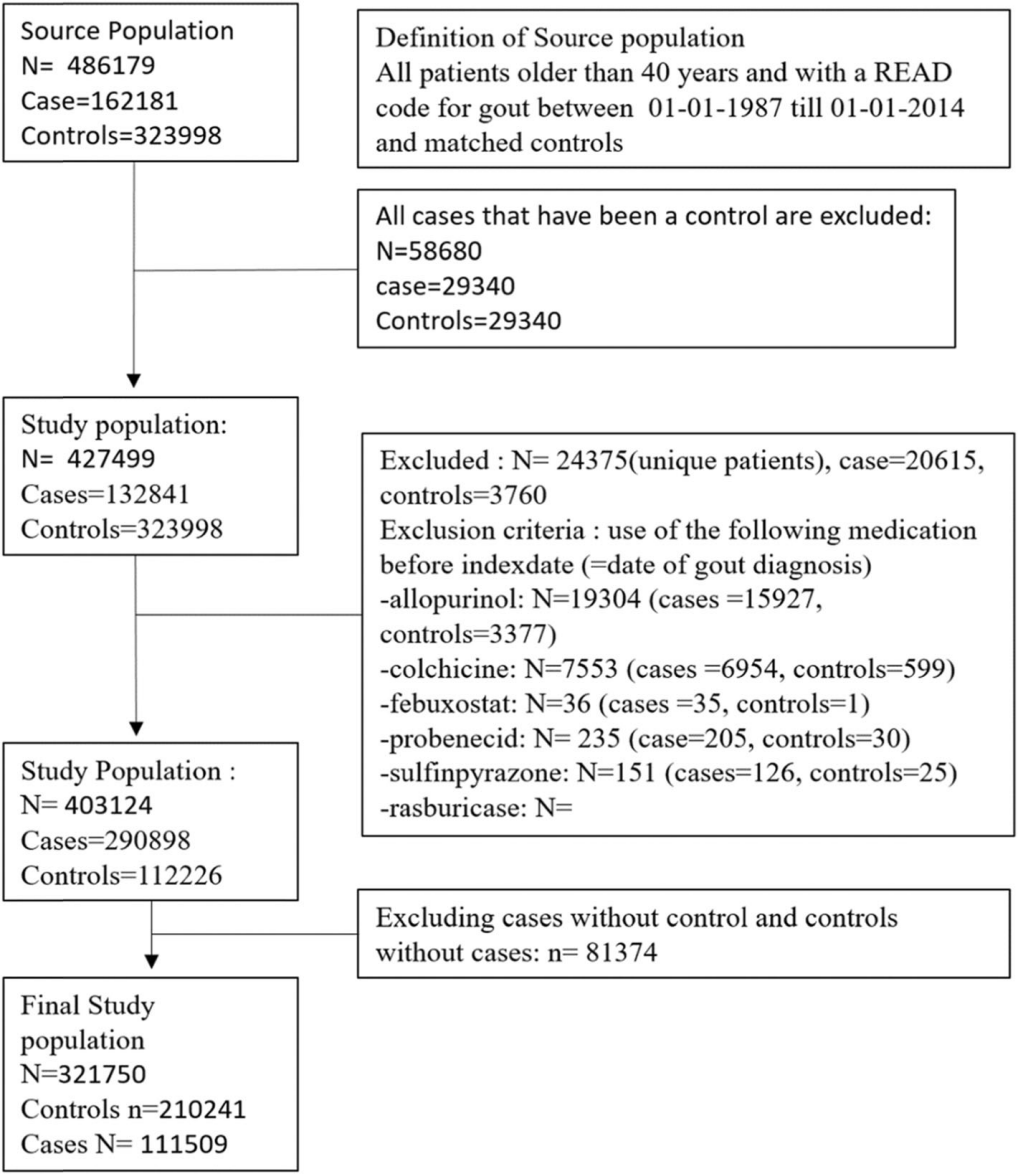
Flow chart, study population

We conducted a population-based case-control study (Fig. 1). The cases consisted of all patients aged 40 years and older with a first diagnosis of gout during the period of valid data collection (from 1 January 1987 to 30 June 2014). Each case with gout was identified using READ codes [17]. READ codes are a set of clinical codes used in primary care in the UK for the registration of clinical diagnosis, processes of care (tests, screening, symptoms, patient administration, etc.), and medication. Each case with gout was matched by year of birth, sex, and practice to up to two randomly selected controls without a diagnosis of gout using incidence density sampling [18]. The date of the first recorded diagnosis of gout defined the index date for the cases and controls were assigned the same index date as their matched case. Cases and controls with a history of exposure to colchicine and uric acid-lowering therapy (ULT) (allopurinol, febuxostat, and/or uricosuric drugs) before the index date as well as their matched case or control were excluded.

### Exposure and potential confounders

Clinical READ codes were used to determine OSA exposure. Cases and controls with a read code for OSA before the index date were classified as being exposed to OSA.

The following variables were considered as potential confounders and were assessed prior to the index date: smoking status, BMI, alcohol use, socioeconomic status, a history of hypertension, diabetes mellitus (as recorded by either a diagnostic code for diabetes mellitus or a history of prescription(s) for anti-diabetic treatment, British National Formulary Chapters 6.1.1 and 6.1.2), hypercholesterolemia, postmenopausal status/hysterectomy, acute myocardial infarction, stroke, or heart failure. The use of the following medication was assessed in the 6 months before the index date: thiazide diuretics, loop diuretics, beta-blockers, calcium channel blockers, angiotensin-converting enzyme inhibitors (ACE-inhibitors), angiotensin II receptor blockers (ARBs), low-dose aspirin, statins, non-insulin antidiabetic drugs (NIADDs), insulin, or benzodiazepines. In addition, the most recent eGFR before the index date was assessed. Electronic lab test data were used to extract the eGFR. Furthermore, when only serum creatinine measurements were available, these were used to estimate the eGFR by the use of the abbreviated MDRD formula (186 × (serum creatinine/88.4)^−1.154^ × (age)^−0.203^ × (0.742 if female)). In addition, we identified diagnostic codes for stages of CKD. When there were multiple records on the same day, the best eGFR was chosen. The following categories were used to stratify for renal function by eGFR: CKD 1 (eGFR > 90 ml/min), CKD 2 (eGFR 60–89 ml/min), CKD 3 (eGFR 30–59 ml/min), CKD 4 (eGFR 15–29 ml/min), and CKD 5 (< 15 ml/min).

### Statistical analysis

Conditional logistic regression was used to estimate the risk of gout associated with a diagnosis of OSA (SAS version 9.4, PHREG procedure). In the analyses, risk was expressed as odds ratios (OR) with corresponding 95% confidence intervals (CIs). Potential confounders were included in the final model if they independently changed the beta-coefficient for OSA by at least 5% or when a consensus about inclusion existed within the team of researchers, supported by clinical evidence from the literature. Missing data of confounders such as BMI, smoking status, alcohol use, and renal function were treated as separate levels using dummy variables. OSA exposure was further stratified by gender, age categories, and the presence of important confounders. Finally, we studied the effect of univariately adding the most important confounders to the main analyses as well as adjusting simultaneously for these confounders.

## Results

Table 1 shows the baseline characteristics of the study population. The cohort encompassed 111,509 gout cases and 210,241 controls with a mean age of 62 years (standard deviation SD 13.3), of whom 27% were female. Gout cases had a higher BMI than controls (29 kg/m^2^ SD 5.3 in cases vs. 26.8 kg/m^2^ SD 4.8 in controls). On average, gout cases used alcohol more often (73.9% in cases vs. 65.5% in controls) and were more likely to be ex-smokers than controls (34.2% cases vs. 26% controls). With regard to comorbidities, gout cases more often had a history of hypertension, heart failure, or reduced renal function. They were also more frequently recent users of diuretics.

**Table 1:** Baseline characteristics of cases and matched controls

Characteristics	Cases		Controls	
	*N* = 111,509	%	*N* = 210,241	%
No. of females	30,461	27.3	58,715	27.9
Age (mean, [SD], years)	62.8	13.3	62.5	13.3
By class				
18–49	22,050	19.8	43,103	20.5
49–59	25,927	23.3	50,025	23.8
60–69	26,127	23.4	48,424	23.0
≥ 70	37,405	33.5	68,689	32.7
Smoking status				
Never	46,790	42.0	86,764	41.3
Current	16,077	14.4	39,182	18.6
Ex	38,146	34.2	54,754	26.0
Missing	10,496	9.4	29,541	14.1
BMI, kg/m^2^ (mean [SD])	29	5.3	26.8	4.8
By category				
< 25	18,938	17.0	60,497	28.8
25–30	39,492	35.4	65,842	31.3
31–34	22,075	19.8	24,369	11.6
≥ 35	10,992	9.9	8925	4.2
Missing	20,012	17.9	50,608	24.1
Alcohol				
No	16,639	14.9	34,934	16.6
Yes	82,405	73.9	137,605	65.5
Missing	12,465	11.2	37,702	17.9
Renal function*				
CKD 1	8382	7.5	18,529	8.8
CKD 2	31,838	28.6	56,712	27.0
CKD 3	19,230	17.2	16,744	8.0
CKD 4	2001	1.8	611	0.3
CKD 5	206	0.2	146	0.1
Missing	49,852	44.7	117,499	55.9
History of comorbidities				
Acute myocardial infarction	7858	7.0	8318	4.0
Stroke	5952	5.3	8283	3.9
Heart failure	8954	8.0	5213	2.5
Hypertension	49,488	44.4	54,166	25.8
Diabetes mellitus	10,928	9.8	16,058	7.6
Hypercholesterolemia	8699	7.8	10,891	5.2
OSA	1094	1.0	1126	0.5
Use of diuretics^+^				
Loop diuretics	17,976	16.1	11,377	5.4
Thiazide diuretics	24,049	21.6	22,012	10.5

Abbreviations: *N* number, *SD* standard deviation, *BMI* body mass index, *CKD* chronic kidney disease, *OSA* obstructive sleep apnea

*CKD 1 (eGFR > 90 ml/min), CKD 2 (eGFR 60–89 ml/min), CKD 3 (eGFR 30–59 ml/min), CKD 4 (eGFR 15–29 ml/min), CKD 5 (< 15 ml/min)

^+^Within 6 months prior to index date

Patients with OSA had an almost doubled risk of gout (crude odds ratio [OR] 1.86; 95% confidence interval [CI] 1.71–2.02, Table 2). However, the effect disappeared after statistical adjustment for alcohol use, a history of diabetes mellitus, renal function, the most recently recorded eGFR measurement, heart failure, smoking status, BMI category, and recent use of statins, beta-blockers, ACE-inhibitors, ARBs, calcium channel blockers, loop diuretics, or thiazide diuretics (adjusted [adj.] OR 1.05; 95% CI 0.96–1.16). Further exploration identified that this shift was almost entirely explained by statistical adjustment for BMI, heart failure, recent use of diuretics, and renal function (Table 3).

**Table 2:** Risk of gout in patients with OSA, stratified by gender, age, BMI, CKD, comorbidities, and recent use of diuretics

Exposure	Cases		Controls		Crude	Fully adj.
	*N* = 111,509	%	*N* = 210,241	%	OR (CI)	OR
No OSA	110,415	99.0	209,115	99.5	Referent	Referent
OSA	1094	0.98	1126	0.54	1.86 (1.71–2.02)	1.05 (0.96–1.16)
By gender						
Male	953	0.85	1043	0.50	1.74 (1.59–1.90)	1.05 (0.95–1.16)
Female	141	0.13	83	0.04	3.36 (2.56–4.42)	1.64 (1.19–2.27)
By age class						
40–49 years	210	0.19	186	0.09	2.22 (1.82–2.71)	1.12 (0.90–1.41)
50–59 years	346	0.31	392	0.19	1.73 (1.50–2.00)	0.96 (0.82–1.13)
60–69 years	316	0.28	341	0.16	1.75 (1.49–2.04)	1.02 (0.86–1.22)
> 70 years	222	0.20	207	0.10	1.96 (1.62–2.37–1.13)	1.20 (0.96–1.50)
By BMI, kg/m^2a^						
< 25	40	0.04	113	0.05	0.67 (0.47–0.97)	0.67 (0.45–0.98)
25–29	224	0.20	305	0.15	1.38 (1.16–1.64)	1.15 (0.95–1.39)
30–34	318	0.29	325	0.15	1.88 (1.61–2.20)	1.34 (1.13–1.59)
≥ 35	456	0.41	322	0.15	2.74 (2.37–3.16)	1.56 (1.33–1.83)
Missing	56	0.05	61	0.03	1.78 (1.24–2.57)	1.91 (1.30–2.81)
By renal function^+,a^						
CKD 1	151	0.14	250	0.12	1.17 (0.95–1.43)	0.61 (0.49–0.76)
CKD 2	492	0.44	527	0.25	1.80 (1.59–2.03)	1.02 (0.89–1.17)
CKD 3	233	0.21	92	0.04	4.76 (3.72–6.07)	2.22 (1.70–2.91)
CKD 4	19	0.02	< 5^#^	0.00	11.24 (3.34–37.81)	3.93 (1.06–14.56)
CKD 5	< 5^#^	0.00	< 5^#^	0.00	1.00 (0.09–11.03)	0.41 (0.04–4.59)
Missing	198	0.18	252	0.12	1.50 (1.24–1.81)	1.15 (0.94–1.40)
By history of comorbidities						
Acute myocardial infarction^a^						
Yes	80	0.07	73	0.03	2.08 (1.51–2.86)	0.80 (0.56–1.15)
No	1014	0.91	1053	0.50	1.84 (1.69–2.01)	1.07 (0.97–1.18)
Stroke^a^						
Yes	52	0.05	42	0.02	2.29 (1.52–2–3.44)	1.05 (0.66–1.66)
No	1042	0.93	1084	0.52	1.84 (1.69–2.01)–2.21)	1.05 (0.96–1.16)
Heart failure^a^						
Yes	128	0.11	37	0.02	6.61 (4.58–9.54)	1.82 (1.21–2.73)
No	966	0.87	1089	0.52	1.70 (1.56–1.85)	1.01 (0.92–1.12)
Diabetes mellitus^a^						
Yes	265	0.24	258	0.12	1.96 (1.65–2.33)	0.70 (0.58–0.85)
No	829	0.74	868	0.41	1.83 (1.66–2.01)	1.16 (1.05–1.30)
Hypertension ^a^						
Yes	659	0.59	466	0.22	2.71 (2.40–3.05)	1.14 (1.00–1.30)
No	435	0.39	660	0.31	1.27 (1.12–1.43)	0.98 (0.86–1.12)
Hypercholesterolemia ^a^						
Yes	553	0.50	521	0.25	2.02 (1.79–2.28)	0.94 (0.82–1.08)
No	541	0.49	605	0.29	1.72 (1.53–1.94)	1.16 (1.02–1.32)
By use of loops diuretics*,^a^						
Yes	260	0.23	91	0.04	5.41 (4.26–6.87)	1.73 (1.33–2.26)
No	834	0.75	1035	0.49	1.55 (1.41–1.69)	1.01 (0.91–1.12)
By use of thiazide diuretics*,^a^						
Yes	274	0.25	129	0.06	4.10 (3.32–5.06)	1.85 (1.47–2.33)
No	820	0.74	997	0.47	1.58 (1.43–1.73)	0.93 (0.84–1.04)

Abbreviations: *N* number, *OR* odds ratio, *CI* confidence interval, *Fully adj.* fully adjusted: adjusted for smoking status, alcohol use, body mass index, history of diabetes mellitus, heart failure and the most recently recorded eGFR measurement. In addition, we adjusted analyses for the use of statins, beta-blockers, Angiotensin-converting enzyme inhibitors, angiotensin II receptor blockers, calcium channel, blockers and thiazide or loop diuretics 6 months before the index date, *OSA* obstructive sleep apnea, *CKD* chronic kidney disease, *BMI* body mass index

^+^By the most recently recorded eGFR prior to index date. CKD 1 (estimated glomerular filtration rate [eGFR] > 90 ml/min), CKD 2 (eGFR 60–89 ml/min), CKD 3 (eGFR 30–59 ml/min), CKD 4 (eGFR 15–29 ml/min), CKD 5 (< 15 ml/min)

^#^According to the Independent Scientific Advisory Committee (ISAC) guidance on the content of protocols for research using CPRD data, no cell containing < 5 cases or controls are reported

*Within 6 months prior to index date

^a^The stratified analysis was not adjusted for the factor by which it was stratified

**Table 3:** Statistical adjustment by body mass index, heart failure, and renal function and the association between OSA and gout

Exposure	Odds ratio
	(95% confidence interval)
No OSA	**Reference**
OSA	
Crude odds ratio	1.86 (1.71–2.02)
Adjusted by	
BMI	1.22 (1.12–1.33)
Most recently recorded renal function	1.61 (1.47–1.75)
History of heart failure	1.77 (1.63–1.93)
Use of thiazide diuretics in previous 6 months	1.69 (1.55–1.85)
Use of loop diuretics in previous 6 months	1.59 (1.46–1.73)
All of the abovementioned confounders	1.05 (0.96–1.16)

Renal Function = renal function was estimated by lab data containing the most recently recorded eGFR. When only creatinine values were available, the MDRD formula was used to calculate the eGFR. In addition, read codes for the stage of chronic kidney disease were used to determine renal function

Abbreviations: *OSA* obstructive sleep apnea, *BMI* body mass index

Stratification of the fully adjusted models (Table 2) revealed that as compared to patients without OSA, those with OSA and with a high BMI remained at an increased risk of gout (BMI 30–34 kg/m^2^—adj. OR 1.34; 95% CI 1.13–1.59; BMI ≥ 35 kg/m^2^—1.56; 95% CI 1.33–1.83). Also, in comparison to patients without OSA, those with OSA and a history of heart failure had an almost doubled risk of gout (adj. OR 1.82; 95% CI 1.21–2.73). Furthermore, recent use of loop diuretics (adj. OR 1.73; 95% CI 1.33–2.26) and use of thiazide diuretics (adj. OR 1.85; 95% CI 1.47–2.33) was also associated with an increased risk of gout. The risk of gout among patients with OSA also further rose with increasing renal impairment (adj. OR 2.22; 95% CI 1.70–2.91 for CKD 3 (eGFR 30–59 ml/min), adj. OR 3.93; 95% CI 1.06–14.56 for CKD 4 (eGFR 15–29 ml/min) (Table 2). With regard to sex, women with OSA remained at an increased risk of gout in contrast to men with OSA (adj. OR 1.64; 95% CI 1.19–2.27).

Compared to patients without OSA, patients with OSA and diabetes mellitus had a statistically significant decreased risk of gout (adj. OR 0.70; 95%CI 0.58–0.85). Patients with OSA and hypercholesterolemia also had a 6% decreased risk, although not statistically significant (adj. OR 0.94; 95%CI 0.82–1.08).

## Discussion

Our study showed that the almost doubled risk of gout with OSA disappeared after adequate statistical adjustment for BMI, renal function, heart failure, and recent use of diuretics. Notwithstanding, subgroups of patients with OSA, more specifically women and those with a history of heart failure, who had recently used diuretics, who had an eGFR between 59 and 15 ml/min, or who had a BMI above 30 kg/m2, still had a 2- to 4-fold increased risk of gout.

**Fig. 2:**
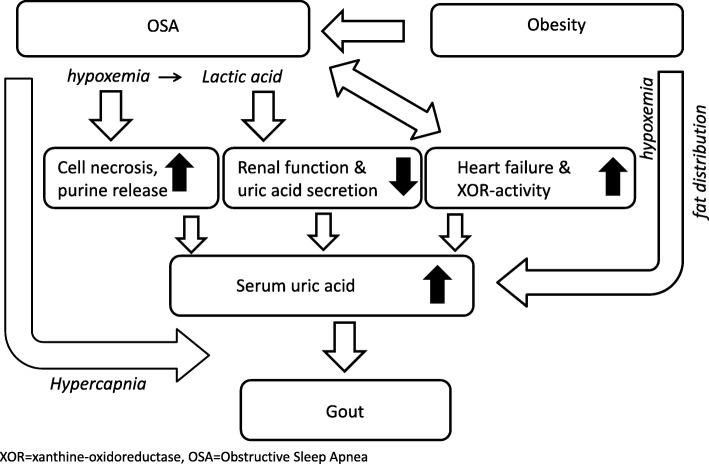
Possible biological pathways linking OSA to gout. XOR, xanthine oxidoreductase; OSA, obstructive sleep apnea

The absence of an overall association between OSA and gout in our study contrasts with a 1.5-fold increased risk of gout with OSA that was reported by two previous studies [4, 9] which had used the same data source, i.e., the CPRD GOLD database, or a data source (THIN) that partly overlaps with CPRD [19]. Table 3 shows that this difference can be largely explained by more comprehensive statistical adjustment for potential confounding in the current study, in particular for renal impairment, as measured by eGFR and READ codes, and heart failure. When renal function declines, less uric acid is excreted which leads to hyperuricemia and eventually gout [20] (Fig. 2).

Heart failure is probably under-recorded in large observational studies based on diagnostic codes [21]. Specific for the UK primary care databases, under-diagnosis might be related to manual coding of cardiology discharge letters by general practice staff. Therefore, we statistically adjusted our analyses for proxy indicators of heart failure such as recent use of diuretics. We also statistically adjusted our analysis for the use of other medications that are commonly used for heart failure, including beta-blockers, ACE-inhibitors, and ARBs [22]. Since (repeated) prescriptions, including outpatient prescriptions of cardiologists, are generally issued by GPs every 4 weeks, this proxy indicator is likely to be better captured and therefore more likely to further reduce the level of residual confounding. Notwithstanding, heart failure remained associated with gout, even after full adjustment. The explanation might be found in insufficient adjustment for the known increased xanthine oxidoreductase (XOR) activity in the myocardium of the failing heart which leads to an elevation of uric acid [23] (Fig. 2). Filippatos et al. demonstrated in their study that hyperuricemia was associated with poor outcomes in patients with heart failure without CKD but not in those with CKD, confirming the hypothesis that hyperuricemia in patients with heart failure could not only be explained by reduced uric acid excretion because of a poor kidney function [24]. Otaki et al. also demonstrated an association between XOR activity and severity and clinical outcomes in patients with heart failure [25]. Further evidence on a possible role of an increased XOR activity in heart failure can be found in studies demonstrating a beneficial effect of adding allopurinol to the treatment of patients with heart failure [26, 27]. This beneficial effect was not demonstrated with benzbromarone, which is a uricosuric drug and therefore decreases the uric acid concentration by increasing its excretion [28]. An alternative explanation for the independent contribution of heart failure to OSA can also be found in the influence of overnight rostral fluid shifts to the neck and lungs in patients with heart failure [29].

Unexpectedly, we found that the risk of gout with OSA disappeared in men after adjustment for confounders, while in women, the risk remained elevated. It is widely accepted that females are at lower risk of gout, as a result of the uricosuric effect of estrogens in women before menopause [30]. However, even after menopause, the risk in females remains lower and we can therefore assume that other causal factors probably play a role. Sex differences have also been noted in the prevalence and severity of OSA, with women presenting with less severe and less prevalent disease. These differences are decreased after menopause [31]. Differences in fat distribution, upper airway anatomy (in particular the posterior tongue region), mechanisms affecting ventilatory stability, and sex hormones might explain the differences between men and women in OSA [31]. In this line, it is of note that a study by Wang et al. showed that fat accumulation around the head measured by dual-energy X-ray absorptiometry was positively correlated with uric acid levels in women but not in men [32].

Obesity, which itself is associated with hypoxemia, is the main risk factor for the development of OSA. In more obese patients, OSA is aggravated with more severe oxygen desaturations and hypoxemia, which may explain why the risk of gout remains high in the highest BMI category [33, 34] (Fig. 2).

Our study had several limitations. First, there probably is underreporting of both OSA and gout, especially in the less severe cases [17, 35]. Misclassification of both exposure (OSA) and our outcome of interest, i.e., gout, is probably random and may therefore lead to regression towards null [36]. It could have masked a true association between OSA and gout among men. Among women, it could have masked a higher true association. With respect to confounders, although our data regarding renal function were more accurate, renal function is not routinely measured in primary care. This could lead to residual confounding. Another limitation in our study, which is present in all epidemiological studies where researchers try to estimate the total causal effect of an exposure on an outcome of interest, is the problem of potential over-adjustment. Ideally, one should not control for factors which lie in the causal pathway between exposure and outcome, as it leads to a regression of the risk towards null [37]. In our case, renal function could also be influenced by OSA itself as nocturnal hypoxemia present in OSA could accelerate the decline in kidney function and therefore reduce uric acid excretion and induce or exacerbate hyperuricemia and eventually the risk of gout [38]. Renal function would then be in the causal pathway from OSA to gout. Another limitation concerns the limited number of patients present in some subgroups, especially women and the groups with the worst CKD (CKD 4 and 5). The conclusion drawn from those results should therefore be interpreted with caution.

Our study had several strengths. First, we were able to include a large number of patients with gout and controls. The findings of this study are therefore likely to be generalizable to patients with gout and OSA in the total UK population [14]. Second, the large amount of clinical information routinely and longitudinally collected in clinical practice allowed us to statistically adjust for many potential confounders such as age, sex, smoking status, alcohol use, kidney function, comorbidity, and use of medication.

## Conclusion/key message

This study showed that the observed association between OSA and gout disappeared after extensively adjusting for BMI, heart failure, diuretics, and renal function, in particular. As the latest guidelines for the treatment of gout by the British Society of Rheumatology recommend to discuss the use of ULT with every patient, even after a first attack of gout, we think that it is important that physicians are aware that gout occurs more frequently in the presence of various comorbidities, among which OSA. Our study also emphasizes the importance of using frequently recorded electronic lab test data to assess renal function in UK primary care data, rather than READ codes.

## Data Availability

The datasets used and/or analyzed during the current study are available from the corresponding author on reasonable request.

## Abbreviations

OSA: Obstructive sleep apnea
BMI: Body mass index
MSU: Monosodium urate
ATP: Adenosine triphosphate
UK: United Kingdom
CKD: Chronic kidney disease
eGFR: Estimated glomerular filtration
CPRD: Clinical Practice Research Datalink
GP: General practitioner
ULT: Uric acid-lowering therapy
ACE-inhibitors: Angiotensin-converting enzyme inhibitors
ARBs: Angiotensin II receptor blockers
NIADDs: Non-insulin antidiabetic drugs
CI: Confidence interval
SD: Standard deviation
OR: Odds ratio
Adj.: Adjusted
